# Love as a Commitment Device: Evidence from a Cross-Cultural Study across 90 Countries

**DOI:** 10.1007/s12110-024-09482-6

**Published:** 2024-12-27

**Authors:** Marta Kowal, Adam Bode, Karolina Koszałkowska, S. Craig Roberts, Biljana Gjoneska, David Frederick, Anna Studzinska, Dmitrii Dubrov, Dmitry Grigoryev, Toivo Aavik, Pavol Prokop, Caterina Grano, Hakan Çetinkaya, Derya Atamtürk Duyar, Roberto Baiocco, Carlota Batres, Yakhlef Belkacem, Merve Boğa, Nana Burduli, Ali R. Can, Razieh Chegeni, William J. Chopik, Yahya Don, Seda Dural, Izzet Duyar, Edgardo Etchezahar, Feten Fekih-Romdhane, Tomasz Frackowiak, Felipe E. García, Talia Gomez Yepes, Farida Guemaz, Brahim B. Hamdaoui, Mehmet Koyuncu, Miguel Landa-Blanco, Samuel Lins, Tiago Marot, Marlon Mayorga-Lascano, Moises Mebarak, Mara Morelli, Izuchukwu L. G. Ndukaihe, Mohd Sofian Omar Fauzee, Ma. Criselda Tengco Pacquing, Miriam Parise, Farid Pazhoohi, Ekaterine Pirtskhalava, Koen Ponnet, Ulf-Dietrich Reips, Marc Eric Santos Reyes, Ayşegül Şahin, Fatima Zahra Sahli, Oksana Senyk, Ognen Spasovski, Singha Tulyakul, Joaquín Ungaretti, Mona Vintila, Tatiana Volkodav, Anna Wlodarczyk, Gyesook Yoo, Benjamin Gelbart, Piotr Sorokowski

**Affiliations:** 1 IDN Being Human Lab - Institute of Psychology, University of Wrocław, Wrocław, Poland; 2 School of Archaeology and Anthropology, Australian National University, Canberra, Australia; 3 Faculty of Educational Sciences, Institute of Psychology, University of Lodz, Łódź, Poland; 4 Macedonian Academy of Sciences and Arts, Skopje, North Macedonia; 5 Crean College of Health and Behavioral Sciences, Chapman University, Orange, CA USA; 6 Humanities Department, Icam School of Engineering, Toulouse Campus, Toulouse, France; 7 Center for Sociocultural Research, HSE University, Moskva, Moscow Russia; 8 Institute of Psychology, University of Tartu, Tartu, Estonia; 9 Department of Environmental Ecology, Comenius University, Bratislava, Slovakia; 10 Department of Psychology, Sapienza University of Rome, Rome, Italy; 11 Department of Psychology, Yaşar University, Izmir, Turkey; 12 Department of Anthropology, Istanbul University, Istanbul, Turkey; 13 Department of Developmental and Social Psychology, Sapienza University of Rome, Rome, Italy; 14 Department of Psychology, Franklin and Marshall College, Lancaster, PA USA; 15 Ecole Normale Supérieure Assia DJEBAR de Constantine, Constantine, Algérie; 16 Department of Psychology, Ege University, İzmir, Turkey; 17 Department of Psychology, University of Georgia, Tbilisi, Georgia; 18 Department of Anthropology, Hatay Mustafa Kemal University, Hatay, Turkey; 19 PROMENTA Research Center, Department of Psychology, University of Oslo, Oslo, Norway; 20 Department of Psychology, Michigan State University, East Lansing, MI USA; 21 School of Education, Universiti Utara Malaysia, Sintok, Kedah Malaysia; 22 Department of Psychology, Izmir University of Economics, İzmir, Turkey; 23 Psicología Evolutiva y de la Educación, Universidad Autónoma de Madrid, Madrid, España; 24 Department of Psychiatry Ibn Omrane, Razi Hospital, Manouba, Tunisia; 25 Institute of Psychology, University of Wrocław, Wrocław, Poland; 26 Departamento de Psiquiatría y Salud Mental, Universidad de Concepción, Concepción, Chile; 27 Department of Education, Universidad Internacional de Valencia, Valencia, Spain; 28 Department of Psychology and Education Sciences and Speech Therapy, Mohamed Lamine Debaghine, Setif2 University, Setif, Algeria; 29 Department of Sosiologie, Ibn Tofail University, Kenitra, Morocco; 30 School of Psychological Sciences, National Autonomous University of Honduras, Tegucigalpa, Honduras; 31 Department of Psychology, University of Porto, Porto, Portugal; 32 Department of Administration, Getulio Vargas Foundation, Rio De Janeiro, Brazil; 33 Escuela de Psicología, Pontificia Universidad Católica del Ecuador- Ambato, Ambato, Ecuador; 34 Department of Psychology, Universidad del Norte, Puerto Colombia, Colombia; 35 Department of Dynamic and Clinical Psychology and Health Studies, Sapienza University of Rome, Rome, Italy; 36 Department of Psychology, Alex Ekwueme Federal University, Ndufu-alike, Nigeria; 37 Faculty of Education and Liberal Arts, INTI International University, Nilai, Malaysia; 38 Department of Psychology, University of Santo Tomas, Manila, Philippines; 39 Department of Psychology, Università Cattolica del Sacro Cuore, Milano, Italy; 40 School of Psychology, University of Plymouth, Plymouth, UK; 41 Department of Psychology, Ivane Javakhishvili Tbilisi State University, Tbilisi, Georgia; 42 Faculty of Social Sciences, imec-mict-Ghent University, Ghent, Belgium; 43 Department of Psychology, University of Konstanz, Konstanz, Germany; 44 Interdisciplinary Sports Science Laboratory, Institute of Sports Professions, Ibn Tofail University, Kenitra, Morocco; 45 WSB Merito University in Gdansk, Gdańsk, Poland; 46 Department of Psychology, Ss. Cyril and Methodius University, Skopje, North Macedonia; 47 Department of Health and Physical Education, Thaksin University, Songkhla, Thailand; 48 School of Education, International University of Valencia, Valencia, Spain; 49 Psychology Department, West University of Timisoara, Timisoara, Romania; 50 Department of Psychology and Pedagogy, Kuban State University, Krasnodar, Russia; 51 Escuela de Psicología, Universidad Católica del Norte, Antofagasta, Chile; 52 Department of Child & Family Studies, Kyung Hee University, Seoul, Republic of Korea; 53 Department of Psychological and Brain Sciences, University of California, Santa Barbara, CA USA; 54 Division of Psychology, University of Stirling, Stirling, UK; 55 Faculty of Medicine of Tunis, Tunis El Manar University, Tunis, Tunisia

**Keywords:** Romantic love, Importance of love, Evolutionary theory, Parental Investment theory, Kephart, Emotion

## Abstract

Given the ubiquitous nature of love, numerous theories have been proposed to explain its existence. One such theory refers to love as a commitment device, suggesting that romantic love evolved to foster commitment between partners and enhance their reproductive success. In the present study, we investigated this hypothesis using a large-scale sample of 86,310 individual responses collected across 90 countries. If romantic love is universally perceived as a force that fosters commitment between long-term partners, we expected that individuals likely to suffer greater losses from the termination of their relationships—including people of lower socioeconomic status, those with many children, and women—would place a higher value on romantic love compared to people with higher status, those with fewer children, and men. These predictions were supported. Additionally, we observed that individuals from countries with a higher (vs. lower) Human Development Index placed a greater level of importance on romantic love, suggesting that modernization might influence how romantic love is evaluated. On average, participants worldwide were unwilling to commit to a long-term romantic relationship without love, highlighting romantic love’s universal importance.

Love is a ubiquitous experience transcending cultural boundaries (Jankowiak & Fischer, 1992; Kowal et al., 2024) and temporal constraints (Hatfield et al., 2012). The nature of love is multifaceted: Humans can feel love for partners, parents, siblings, relatives, friends, other people, pets, and even god (Machin, 2022). In the present work, we focus specifically on romantic love, that is, the love felt for a partner within the context of a romantic relationship (Graham, 2011). Romantic love is commonly divided into at least two subtypes: Passionate love, felt very intensely and experienced most commonly at the beginning of the romantic relationship, and companionate love, which is felt less intensely and experienced most commonly at later stages of the relationship (Walster & Walster, 1978).

A number of theoretical frameworks have been developed to explain love’s existence and underlying function. For instance, some scholars have suggested that romantic love emerges from attachment mechanisms (Hazan & Shaver, 1987; Mikulincer & Shaver, 2018; Shaver et al., 1996), initially forming between infants and caregivers (Bowlby, 1979) before later being co-opted into adult romantic relationships (Bode, 2023). Fisher et al. (2006) have posited that romantic love is one of the primary brain systems that evolved to maintain the pair bond for the purpose of reproduction. In a similar vein, other scholars have postulated that love is an adaptation designed to motivate behavioral commitment (Fletcher et al., 2015; Frank, 1988).

The concept of love as a commitment device was first proposed by Frank (1988) and was later elaborated by Fletcher et al. (2015). This perspective, rooted in evolutionary theory, suggests that love is designed to down-regulate interest in available alternatives and signal this reduction in interest to a partner, motivating commitment to one’s relationship. Human ancestors who signaled romantic love are hypothesized to have more frequently formed enduring pair bonds with their romantic partners than those who did not (Bales et al., 2021), which might have maximized their reproductive success. Thus, the propensity to feel romantic love proliferated to become a universal (or near-universal) human experience (Jankowiak & Fischer, 1992; Kowal et al., 2024).

Romantic love played (and still plays) a crucial role in the provision of psychological and emotional resources, caregiving, increased fidelity, sharing of resources, and co-parenting (Bode & Kushnick, 2021; Sorokowski et al., 2017). Romantic love might be a glue that holds partners together and helps them overcome life obstacles (Coleman, 1988). If, as Frank (1988) argued, romantic love is missing, partners might not stay faithful to each other. Once a better mate becomes available, a purely rational agent would pursue the new and more desirable partner. However, romantic love, in motivating irrational disinterest in romantic alternatives, can reassure one’s partner of their mutual commitment and signal a willingness to stay together through thick and thin (Buss, 2019).

If the primary function of romantic love is to promote commitment, then romantic love should be universally preferred when selecting a long-term partner. Cross-cultural evidence supporting this notion comes from Buss’s (1989) seminal study, which surveyed over 10,000 participants from 33 countries, asking them to rank the importance of 18 characteristics in a long-term mate. Among both women and men, love was rated as the most important characteristic in a mate (Buss et al., 1990).

Romantic love’s function to assure partners of one’s dedication and faithfulness might become especially crucial when times are challenging. Tan et al. (2020) suggested that romantic commitment might be particularly important for individuals of lower socioeconomic status (SES) because they have fewer material incentives with which to secure their social partners. Moreover, those with fewer resources may require resource provisioning from a partner more urgently. Committed partners provide support that alleviates stress (Bolger & Amarel, 2007), which might be more commonly experienced by people of lower SES (Marmot, 2007). Low SES individuals also experience additional stressors that can lead to conflict and higher rates of divorce, increasing the importance of love in maintaining the pair bond during challenging economic situations (Harsoyo & Darmawan, 2023; Karney, 2021; Raz-Yurovich, 2012).

Additionally, the signaling function of love as a commitment device might be particularly crucial for women. According to parental investment theory (Trivers, 1972; for a review, see Mogilski, 2021), males and females incur varying reproductive costs, leading to sex differences in mating strategies. Women bear considerable biological burdens associated with childbearing, including the protracted and costly processes of pregnancy, childbirth, and lactation. By contrast, men may contribute as little as providing genetic material through sperm donation. Given the potential losses that women face if their partner leaves them (and their offspring), human females are hypothesized to be both more selective when choosing romantic partners relative to men (Kanin et al., 1970; Knox & Sporakowski, 1968) and more skeptical of men’s displays of commitment (Haselton & Buss, 2000). Thus, if love acts as a signal of commitment, women might value romantic love more than men.

Finally, the signaling function of love might be particularly important for those with multiple children. Having children together forms strong bonds between partners (Bellido et al., 2013; Onyishi et al., 2012). Thus, the likelihood of divorce is inversely correlated with the number of children (Bellido et al., 2013; Xu et al., 2015), despite evidence suggesting that having children often adversely affects marital satisfaction (Bogdan et al., 2022; Kowal et al., 2021). Moreover, parenting more children tends to be more demanding (Vigouroux & Scola, 2018), creating a greater need for support from a partner (Feinberg, 2003). A deeply committed and loving partner might come to the rescue when a parent’s resources are depleted; this help and care for one’s partner is motivated by romantic love (Sternberg, 1986). Thus, individuals with more children may value love more.

To test these possibilities, we relied on Kephart’s (1967) question, which assesses the importance of romantic love when considering a marriage partner. As a social institution, marriage is recognized across all cultures and is universally associated with a long-term romantic commitment between individuals (Bethmann & Kvasnicka, 2011; Grossbard-Shechtman, 2019; Karney & Bradbury, 2020). This type of long-term commitment is precisely what romantic love is hypothesized to have evolved to support (Fletcher et al., 2015).

Prior research employing Kephart’s question has primarily focused on sex differences in the importance of romantic love, yielding conflicting results. Four studies have supported the finding that men value romantic love more highly than women, with varying effect sizes (Cohen’s *d* = 0.88 [large] in Sprecher & Toro-Morn, 2002; Cohen’s *h* = 0.85 [large] in Kephart, 1967; h = 0.16 [very small] in Simpson et al., 1986; h = 0.03 [very small] in Pavlou, 2009). In contrast, three studies found the opposite, indicating that women value romantic love more than men (averaged Cohen’s *h* = − 0.52 [medium] in Sprecher et al., 1994; d = − 0.19 [very small] in Sprecher & Hatfield, 2017; h = − 0.13 [very small] in Allgeier & Wiederman, 1991), while one study reported no significant sex difference (*d* = 0 in Adamczyk, 2019).

Additionally, three studies have documented cross-cultural differences in the perceived importance of romantic love when considering long-term romantic relationships (Levine et al., 1995; Sprecher et al., 1994; Sprecher & Toro-Morn, 2002). These cross-cultural differences may be partially explained by varying levels of country-level modernization. Previous research has provided evidence that romantic love may be more highly valued in more modernized countries (Baumard et al., 2022; Sorokowski et al., 2023). To account for this, we included the Human Development Index (HDI; United Nations, 2023) as a control variable in our analyses. A detailed summary of existing studies using Kephart’s question can be found in Table S1 in the Supplementary Materials (SM).

To examine sex differences in the importance of romantic love and advance our understanding of the factors potentially explaining intra-individual differences in romanticism, we conducted a cross-cultural study on individuals from 90 countries. Drawing from love as a commitment device perspective, we hypothesized that when considering a long-term romantic relationship (i.e., marriage or registered partnership):

- **H1.** Individuals of lower SES value romantic love more than those of higher SES.
- **H2.** Women value romantic love more than men.
- **H3.** There is a positive relationship between the importance of romantic love and the number of children.

## Material and Methods

The study’s procedure received approval from the first author’s Institutional Review Board (IRB) at the Institute of Psychology, University of Wrocław. Before collecting data, all team members either received ethical approval from their local IRBs or acted on the ethical approval of the first author’s IRB. All participants provided informed consent prior to participating in the survey. All data, R script, and Supplementary Material have been made publicly available at the OSF and can be accessed at https://osf.io/kw2h9.

### Participants

In total, 118,715 participants from 175 countries agreed to complete the survey in one of the 43 languages available. In the subsequent analyses, we included only data from participants who passed the attention check, were from countries with a minimum sample size of 30 individuals per country (Arend & Schäfer, 2019; Lieberoth et al., 2021), had no missing data on the main variables of interest, and reported being either women or men. The final sample included 86,310 individuals from 90 countries, among whom 58,195 (67%) were women and 30,326 (35%) were students. Ages ranged from 18 to 90 (*M* = 30.11, *SD* = 12.32). Detailed demographic profiles for each country can be found in Table S2 in the SM.

### Procedure

We utilized a forward-back translation process (Kowal, 2024) to translate the survey into 45 linguistic versions, allowing people from diverse linguistic backgrounds to comfortably participate in our study. Each of the translation teams was provided with detailed instructions, available openly on the OSF (https://osf.io/kw2h9). Upon completion of the translation, data collection started in April 2021 and ended in August 2021. Most data were collected online, except in Algeria and Morocco, where collaborators used the paper-pencil method. The samples were pooled from diverse sources (such as social media, university mailing lists, newspapers, local community groups, and word-of-mouth advertising), which enabled us to include individuals of different ages, genders, and socioeconomic backgrounds (e.g., residents of small and large cities, community and university samples).

### Measures

For the present analyses, we used the following measures:

#### Importance of Love

To examine the importance of romantic love for long-term romantic relationships (Kephart, 1967), we asked participants the following question: “Assume you are currently not in a committed relationship. Imagine meeting a person who has all of the qualities you desired but who you aren’t in love with. How likely would you be to marry this person/register your partnership with this person?” The response scale ranged from 0 (I would definitely not marry this person) to 100 (I would definitely marry this person), which we reverse-coded so that higher values indicated more importance placed on romantic love.

#### Demographics

Participants were asked to self-report their SES by answering the question, “How good are your financial prospects?” Responses were indicated on an 11-point scale, ranging from 1, “*Extremely poor financial prospects (Bottom 1 out of 100 people)*” to 11, “*Extremely good financial prospects (Top 1 out of 100 people).*” Participants indicated their gender by choosing one of the following options: Male, Female, Nonbinary/Third gender, or “Prefer not to say.” The number of children a participant could report ranged from “0” to “5 or more.”

#### Human Development Index (HDI)

We used the Human Development Index (HDI; United Nations, 2023) as a composite statistic for measuring and comparing levels of development between countries. HDI combines the nation’s longevity (life expectancy at birth), education (mean years of schooling completed at 25 years old and years of schooling expected for a child), and income (Gross National Income per capita); it is a frequently used proxy of countries’ modernization level (e.g., Sorokowski et al., 2023).

### Statistical Analyses

In the first step, Pearson correlations of the main variables were computed. Normality of the variables was investigated using commonly recommended cutoffs of univariate kurtosis values no larger than |7| and skewness values no larger than |2| (Kim, 2013). To detect potential outliers, the Mahalanobis Distance for the variables of interest was calculated using a cutoff of *p* < .001. Individual-level SES and the number of children were country-mean centered, and HDI was grand-mean centered. The importance of the love variable was reverse-coded so that higher scores represented more importance placed on love.

In the next step, multilevel models were conducted, with the importance of love score as the outcome variable and participants nested within the countries. The first null model included only the intercept. The second model introduced the predictor variables, including country-level HDI, individual-level SES, dummy-coded gender (with men as a reference category), and the number of children. In the third model, individual-level slopes were freed. The models were then compared using the Bayesian Information Criterion (BIC) and Akaike Information Criterion (AIC), with a better fit being suggested by changes in the BIC and AIC between the two models exceeding 10 (Burnham & Anderson, 2004; Raftery, 1995). All analyses were performed in R (version 4.3.1).

## Results

**Fig. 1:**
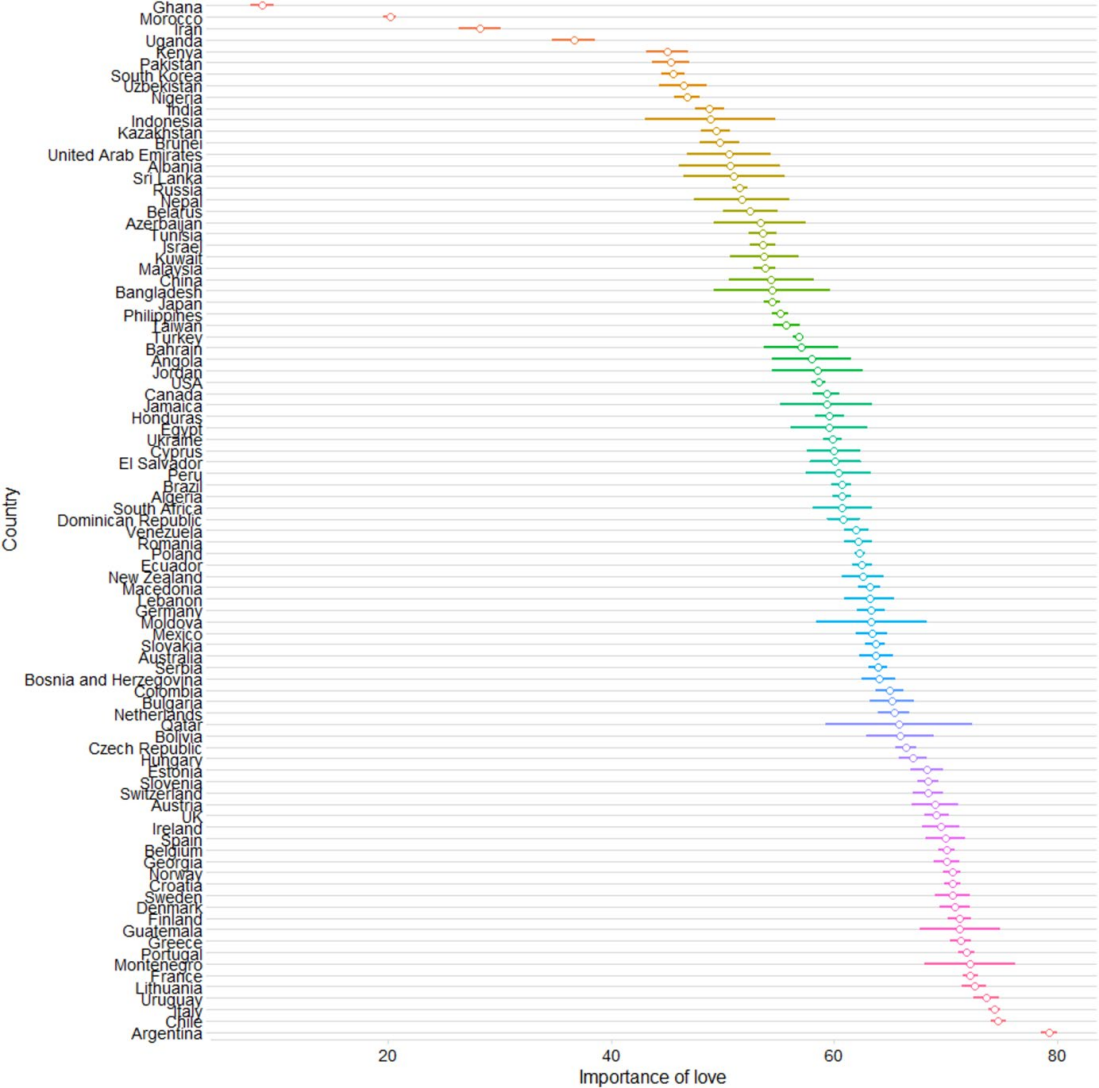
Mean ratings of the importance of romantic love when considering a long-term romantic relationship across countries (error bars represent standard errors)

Figure 1 presents the average scores for the importance of love when considering a long-term romantic relationship across countries. The Pearson correlations between the variables of interest are shown in Table S3 in the SM. All the variables, except for the number of children, were within the expected range of kurtosis and skewness values. The number of children variable had one unit added and was log-transformed, which improved skewness values (from 2.082 to 1.490). However, because the pattern of results was virtually the same and the differences in the coefficient values were marginal, we decided to retain the original number of children variable in all analyses. Similarly, the Mahalanobis Distance inspection suggested that data from 949 individuals might be considered outliers, but analyses with the data included and excluded yielded the same pattern of results. Thus, the analyses we report herein are performed using the complete dataset, without excluding any outliers.

When comparing the BIC and AIC, the second model had a better fit than the first (ΔBIC = 38555, ΔAIC = 38592), and the third had a better fit than the second (ΔBIC = 299, ΔAIC = 383). Hereafter, we present the results of the third model (for BIC and AIC of all models, see Table S4 in the SM). However, it is noteworthy that the second and third models yielded a nearly identical pattern of results.

Table 1 presents the results of the multilevel analysis. Across nearly all the countries in our sample, participants highly valued romantic love when considering a long-term romantic relationship (Fig. 1). Support was found for all three hypotheses. Individuals of lower SES valued romantic love more than those of higher SES (**H1**). Romantic love was more important for women than for men (**H2**). The more children participants had, the more value they placed on romantic love (**H3**). Additionally, individuals from countries with higher HDIs valued romantic love more than those from countries with lower HDIs.

**Table 1:** Results of the multilevel model with the importance of romantic love when considering a long-term romantic relationship as an outcome variable

Fixed effects	β	SE	95% CI	*p*
HDI	0.128	0.022	[0.084, 0.172]	< 0.001***
SES	–0.043	0.007	[–0.056, − 0.029]	< 0.001***
Gender	0.240	0.016	[0.208, 0.271]	< 0.001***
Children	0.027	0.005	[0.017, 0.038]	< 0.001***
Random effects	***Variance***	***SD***		
Intercept	0.068	0.261		
SES	0.002	0.047		
Gender	0.014	0.117		
Children	0.001	0.032		
Residual	0.863	0.929		

* *p* < .05, ** *p* < .01, *** *p* < .001. *ICC* = 0.073, Pseudo *r*^*2*^ = 0.035, *df*_*residuals*_ = 86,294, deviance = 232798.2

**Fig. 2:**
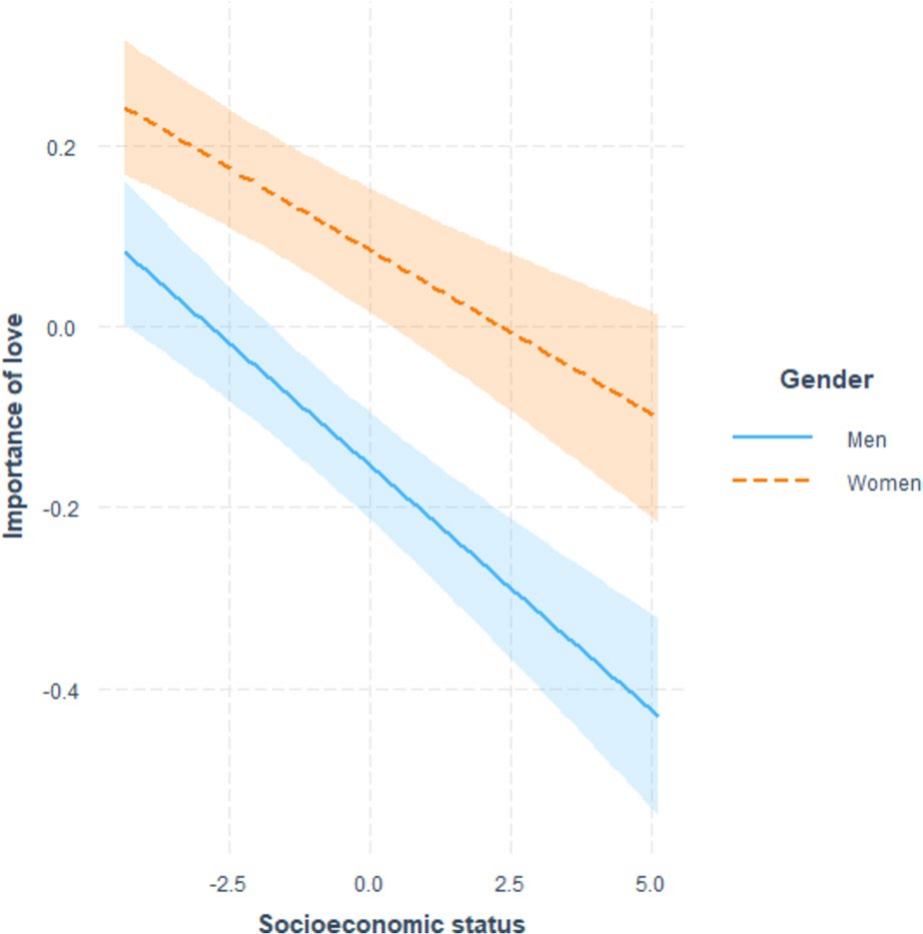
The interaction between the importance of love when considering a long-term romantic relationship and SES across men and women (shaded areas represent 95% confidence intervals)

Because the effect of gender yielded the largest effect size, we followed up the analyses with three models: One with the interaction terms with gender introduced, one for women only, and one for men only. The only significant interaction was with SES (Fig. 2). Importantly, the pattern of results was the same for both genders (for details, see Tables S5 and S6 in the SM). We also investigated the gender differences in the importance of love when considering a long-term romantic relationship within countries by computing Cohen’s *d* values (see Table S7 and Figure S1 in the SM). Across countries, the average *d* value for the observed gender difference was –0.26. Only in Morocco and Tunisia did men value romantic love more than women.

In the final step, we tested the robustness of the negative association between SES and the importance of romantic love in the context of long-term relationships by re-running the analyses using an alternative self-reported measure of SES: social class. Participants responded to the question “Which of the following best describes your social class level?” with five possible answers: upper class (1), upper middle class (2), middle class (3), lower middle class (4), and lower class (5). The responses were reverse-coded, with higher values indicating higher social class. The results mirrored our initial findings (for details, see Table S8 in the Supplementary Materials).

## Discussion

In the present study, we tested hypotheses derived from the concept of love as a commitment device (Fletcher et al., 2015; Frank, 1988), which suggests that romantic love evolved as an adaptive mechanism that aids in maintaining a pair bond, thus enhancing lovers’ reproductive success. Based on the analysis of 86,310 individuals across 90 countries, we observed that, when considering a long-term romantic relationship, romantic love was highly valued in nearly all the countries in our sample. Moreover, romantic love was particularly important for individuals of lower (vs. higher) SES (Hypothesis 1), women (vs. men; H2), and those with more (vs. fewer) children (H3). Additionally, we found evidence that when considering a long-term romantic relationship, romantic love was more important for participants from more (vs. less) modernized countries.

Our findings highlight love’s role as a potent commitment mechanism with diverse implications for maintaining strong bonds within partnerships, albeit with important variation across cultures. Prior research provided evidence that even committed individuals may exhibit attentional bias toward attractive others (Ritter et al., 2010; Simpson et al., 1990), and romantic love priming is enough to suppress such thoughts of attractive alternatives (Gonzaga et al., 2008; Ma et al., 2015). Furthermore, romantic partners express their love and reassure their commitment to each other through nonverbal cues (Gonzaga et al., 2001). Romantic partners desire emotional and physical union (Sternberg, 1986), sometimes achieving it by including the partner in the cognitive self (Branand et al., 2019) or perceiving bodily overlap with the partner (Quintard et al., 2021). Finally, people worldwide indicated that romantic love is a crucial aspect of long-term romantic relationships, thus echoing the results of a cross-cultural study from almost half a century ago in which romantic love was found to be a critical mate preference (Buss, 1989).

According to the concept of love as a commitment device, romantic love acts as a cohesive force that binds partners together. Thus, when considering a long-term romantic relationship, romantic love is expected to be more important for individuals who have potentially more to lose in the event of romantic relationship dissolution, such as those from lower socioeconomic backgrounds, who may face difficulties in maintaining financial stability when left by their partners (Conger et al., 1997; McLanahan & Sandefur, 2009; Vyas & Dillahunt, 2017). By contrast, individuals from higher socioeconomic backgrounds are typically more satisfied with their income (Vera-Villarroel et al., 2015; Ward & King, 2019) and have more extensive economic resources to support themselves (Marmot, 2007). They are also more desirable as future spouses (for a review, see Shafer & James, 2013), perhaps due to more positive self-regard (Renger et al., 2024). Our study corroborates this perspective by finding evidence for a negative association between the importance of romantic love when considering a long-term romantic relationship and individual-level SES, though the strength of this association was not large.

Conversely, we observed a positive relationship between the importance of romantic love when considering a long-term romantic relationship and a proxy of country-level SES—the Human Development Index. This result, the second strongest observed in the present study, is fascinating since it runs opposite to what we observed on the individual level (i.e., participants’ SES). However, evolutionary scholars emphasize the impact of environmental conditions and cultural contexts on human cognition, behaviors, and emotions, leading to significant variations in ostensibly universal traits (Lewis et al., 2021). Thus, although romantic love is recognized as a culturally universal phenomenon (Jankowiak & Fischer, 1992; Kowal et al., 2024) and is hypothesized to have evolved to facilitate pair bonding and enhance reproductive success (Buss, 2019), cultural influences may nonetheless also shape evaluations of romantic love’s importance (Cullen, 2022). Given that mass media and popular culture in more modernized countries often promote romantic love as a fundamental life goal (Dukes et al., 2003; Hefner & Wilson, 2013), it is unsurprising that individuals immersed in such cultural narratives value romantic love particularly highly. Interestingly, the ideal of romantic love depicted in mass media has also been extended to encompass not just one but multiple objects of love, with polyamory serving as one example (Hurson, 2016).

There is a common belief that men are more romantic than women (Orbuch, 2009), and empirical research has provided some support for this assertion. For instance, men typically score higher on the Romantic Beliefs Scale than women (Sprecher & Metts, 1989). Men also tend to fall in love and say “I love you” faster than women (Bode et al., 2024; Harrison & Shortall, 2011; Watkins et al., 2022). However, women place stronger emphasis on emotional connection than men (Buss, 1995; Shackelford & Buss, 1997).

According to parental investment theory (Trivers, 1972), these differences can be explained by considering the potential gains and losses an individual faces upon entering a romantic relationship. Initiating a relationship usually results in securing sexual access to the partner (Kislev, 2021). From an evolutionary perspective, this outcome is a highly desirable goal for men, carrying more potential benefits and fewer risks than for women, who, unlike men, face the possibility of becoming pregnant and incurring the high metabolic costs associated with pregnancy and lactation. Men’s experience and expression of romantic love might signal their commitment, reassuring women about the durability of their relationship with their partners. Here, we predicted and found that women also valued romantic love more than men, with this association being the strongest among all the predictors in the present study.

From an evolutionary point of view, the ultimate goal of pair bonding is to facilitate the transmission of genes to subsequent generations (Buss, 2023). Romantic love may help accomplish this objective through initial sex drive, attraction, and pair bonding (Bode, 2023; Fisher, 1998). Once the objective is achieved and a romantic couple has children, nurturing them requires a substantial amount of energy and resources (Maroto, 2018), and as a result, the presence of both parents may be particularly crucial. This need might explain the greater emphasis on romantic love among parents of more children, as observed in our study. However, it should be noted that this association was the weakest among all our predictors. Previous studies conducted on populations inhabiting environments believed to more closely approximate human ancestral conditions suggest that children’s survival rates are higher when both parents contribute to their provision (Winking et al., 2011), with the effects of maternal care naturally higher than paternal care (for a review, see Sear & Mace, 2008).

It is important to note that romantic love ideals do not necessarily reflect actual feelings of love (Sternberg, 1986). Almost everyone—not only lovers—may view romantic love as crucial and associate it with long-term relationships (Mengzhen et al., 2024). However, numerous factors influence the actual experience of romantic love (Machin, 2022). Take SES as an example. Although individuals of lower SES may perceive romantic love as more important than those of higher SES, actual experiences of romantic love may be more intense among individuals of higher SES. Everyday challenges and financial struggles encountered by individuals of lower SES can contribute to increased conflict between partners and, in turn, hinder romantic love feelings (Neff & Karney, 2017).

Some studies have provided support for the notion that romantic love ideals and actual love experiences are distinct phenomena. For instance, Holmberg and MacKenzie (2002) found that people’s beliefs about how romantic relationships should unfold were unrelated to their actual experiences of romantic love. Other researchers have provided preliminary evidence that romantic scripts can influence actual love feelings, though through different mechanisms. On one hand, romantic beliefs may positively affect the intensity of love, particularly when the relationship is fulfilling (Soyer & Sünbül, 2023). On the other hand, reflecting on discrepancies between romantic ideals and actual experiences of love may lead to negative emotions and dissatisfaction with one’s romantic relationship (Metz, 2007). This possibility helps contextualize the present results alongside previous findings; it offers another potential explanation for why individuals of lower SES, despite valuing romantic love more highly than those of higher SES, might still experience lower levels of romantic love feelings (Neff & Karney, 2017).

While our study provides novel insights into the concept of romantic love as a commitment device, it is essential to acknowledge several limitations that are common in cross-cultural research (Kowal et al., 2022; Sorokowski et al., 2023). First, the sample predominantly consisted of well-educated individuals, which does not fully represent the diverse populations of the countries included. Therefore, despite collecting data from a broad selection of countries, caution must be exercised in generalizing the results to all human cultures. Furthermore, we acknowledge that our assessment of SES primarily focused on resource capital, omitting other important dimensions, such as educational and cultural capital (for a discussion of different approaches to measuring SES, see Avvisati, 2020). Our primary analyses included assessments of financial prospects. These prospects are not limited to the current possession of wealth but reflect a capacity to acquire resources. Prior research has shown that ambition and industriousness can serve as important indicators of future wealth (Buss & Schmitt, 2019). However, the latter two items may also capture one’s optimism. As a robustness check, we conducted a follow-up analysis using another SES measure: participants’ self-reported social class. Importantly, the pattern of results remained consistent. While the distribution of self-reported SES in our sample was normal, individuals from lower SES backgrounds may still be underrepresented. Future research could benefit from the use of more comprehensive SES measures, such as income, to capture participants’ socioeconomic standing more precisely.

Second, we relied on a single-item question about the importance of romantic love when considering a long-term relationship (Kephart, 1967), and thus, standard measures of testing the scale’s reliability cannot be applied (Cronbach, 1951). However, the rising popularity and acceptance of single-item measures underscore their utility (Jovanović & Lazić, 2020), with some scholars arguing that single-item measures perform comparably well to multi-item scales (Niehuis et al., 2024). Third, while Kephart’s (1967) question serves as one approach to assessing the importance of romantic love when considering a long-term romantic relationship, future research could employ alternative measures, such as the Romantic Beliefs Scale (Sprecher & Metts, 1989). Fourth, Kephart’s (1967) question employed in the present study was framed within the context of marriage and registered partnership. The institution of marriage is universally recognized (Bethmann & Kvasnicka, 2011; Grossbard-Shechtman, 2019; Karney & Bradbury, 2020), but considering the gradual decline in the importance of such relationships (Pew Research Center, 2010), some participants might have opposed the idea of engaging in marriage or a registered partnership and not the importance of romantic love in a long-term committed relationship per se. Such an attitude would result in the same pattern of responses in both cases: Not being eager to engage in a loveless marriage, regardless of the underlying reason. However, we deem this possibility unlikely, given that participants from Norway and Sweden—countries with relatively low marriage rates—reported preferences for romantic love which mirrored the results from participants in countries with comparable HDI scores but higher marriage rates (United Nations, 2019). Fifth, because we asked participants a hypothetical question, we cannot draw definitive conclusions about how they would behave in real-life scenarios or even if they all understood the question in the same way.

Sixth, our focus was limited to a small number of factors that might explain differences in the perceived importance of romantic love in long-term relationships. It would be valuable to explore additional socio-cultural and demographic variables, such as relationship type, age, religion, cultural norms around romantic ideals, and attitudes toward marriage and divorce. For example, participants from Ghana, Morocco, and Iran placed the least emphasis on romantic love in the context of long-term relationships. Potential reasons for this may include cultural and religious influences. Specifically, participants from these countries had the highest scores on collectivistic values across all studied countries (with the highest averages in Ghana and Morocco, followed by Angola and Iran). Additionally, these countries also had the highest percentage of Muslim participants (with Morocco having the highest percentage, followed by Iran and Ghana). The historical prevalence of arranged marriages in these regions might also partly explain the observed findings (Parkin, 2021). However, if this were the primary factor driving the results, we would expect participants from India, where arranged marriages are highly prevalent (Jaiswal, 2014), to rate romantic love as least important compared to other countries. However, this was not the case. Other potential explanations for cross-cultural differences such as these warrant further investigation by cross-cultural scholars, who may be interested in utilizing our publicly available data (which can be found on the OSF: https://osf.io/kw2h9).

In summary, our results provide evidence that romantic love is universally recognized as an important factor for long-term romantic relationships, supporting the concept of romantic love as a commitment device. We observed that, when considering a long-term relationship, romantic love was valued more highly by individuals for whom the endurance of their relationship might be more critical or who may face greater losses upon relationship dissolution—namely individuals of lower SES, women, and those with more children. We also found that romantic love was more important for individuals from more modernized countries, which aligns with previous studies suggesting the influence of culture on perceptions of romantic love (Baumard et al., 2022; Sorokowski et al., 2023). In conclusion, our findings underscore the role of romantic love as a pivotal commitment mechanism, shedding light on both its universal significance and cultural variability. Our study highlights its heightened importance among individuals facing socioeconomic challenges, gender disparities, and familial responsibilities while also revealing intriguing patterns across different national cultures.

## Data Availability

All data, R script, and Supplementary Material have been made publicly available at the OSF and can be accessed at https://osf.io/kw2h9.
